# *In vitro* sensitivity of *Plasmodium falciparum* clinical isolates to 4-aminoquinolines in Northeast Nigeria

**DOI:** 10.5281/zenodo.10818088

**Published:** 2016-07-29

**Authors:** Sulayman T. Balogun, Umar K. Sandabe, Isah A. Waziri, Justus Jibrin, Fatai A. Fehintola

**Affiliations:** 1 Department of Clinical Pharmacology and Therapeutics, College of Medical Sciences, University of Maiduguri, Nigeria; 2 Department of Veterinary Physiology, Pharmacology and Biochemistry, Faculty of Veterinary Medicine, University of Maiduguri, Nigeria; 3 Bauchi State College of Health Technology, Ningi, Bauchi State, Nigeria; 4 Department of Pharmacology and Therapeutics, College of Medicine, University of Ibadan, Nigeria

## Abstract

**Background:**

Widespread dr ug-resistant *Plasmodium falciparum* strains have challenged the pivotal role played by 4-aminoquinolines, including chloroquine (CQ), which has been delisted for the treatment of malaria in most parts of the world. This study assessed the *in vitro* sensitivity of *P. falciparum* clinical isolates (PfCIs) to amodiaquine (AQ) and CQ in Northeast Nigeria.

**Materials and methods:**

PfCIs were collected from subjects with uncomplicated *P. falciparum* malaria in Azare, Bauchi State and Maiduguri, Borno State following an informed consent. The *in vitro* sensitivity was assessed by micro-test (Mark

III) method and the IC_50_ of AQ and CQ was determined using HN-NonLin Version VI.1 software. The reference standard cut-off values for *in vitro* AQ and CQ resistance of 80 and 160 nmol/l, respectively, were used. Isolates that were inhibited by lower AQ and CQ concentrations were referred to as sensitive.

**Results:**

Valid *in vitro* assay ^r^ esults were obtained for 88.9% (80/90) of the PfCIs; Azare had 93.3% (28/30) and Maiduguri had 86.7% (52/60) [χ2 = 0.35; df = 1; p = 0.486]. The geometric mean (GM) IC50 of AQ and CQ were 24.2 nmol/l (95% CI, 10.5 – 49.6 nmol/l) and 39.5 nmol/l (95% CI, 34.5 – 49.6 nmol/l), respectively. The AQ (p = 0.922) and CQ (p = 0.085) GM IC50 were similar between Azare and Maiduguri PfCIs. Only one isolate showed *in vitro* resistance to AQ giving a sensitivity of 98.8% (79/80) while 17 PfCIs showed *in vitro* resistance to CQ giving a sensitivity of 78.8% (63/80). The CQ sensitivity was similar between Azare (67.9%; 19/28) and Maiduguri (84.6%; 44/52) PfCIs (χ^2^ = 3.05; df = 1; p = 0.081).

**Conclusions:**

The findings may suggest that the AQ *in vitro* sensitivity remains high and the isolates in Northeast Nigeria may appear more sensitive to CQ than isolates from other parts. These findings may affect malaria treatment and control policy in Nigeria.

## 1 Introduction

Antimalarial chemotherapy is a vital component of malaria treatment and control [1,2] partly because the quest for malaria vaccines is yet to yield convincing results [3]. The 4-aminoquinolines, such as amodiaquine (AQ) and chloroquine (CQ), have had massive contributions to the fight against malaria. CQ was the first-line drug in the treatment of uncomplicated malaria in Nigeria until about a decade ago [4-6]. CQ has a good safety profile and is readily available and affordable. However, the widespread occurrence of CQ-resistant *Plasmodium falciparum* strains in Nigeria [7,8] led to the adoption of the current artemisinin-based combination therapies (ACTs) in 2004 [9] of which AQ, another 4-aminoquinoline, is a major component. In Nigeria today, artemether-lumefantrine and artesunate-amodiaquine are the first and second-line ACTs for the treatment of acute uncomplicated malaria [3,9] and they remain highly effective in Northeast Nigeria [10].

As a key component of effective and sustainable malaria control the World Health Organization (WHO) recommends monitoring of antimalarial sensitivity using *in vivo* (clinical) and *in vitro* methods. In addition, these methods could be complemented by genotyping of resistance biomarkers [11]. This becomes necessary because irrational antimalarial use and *P. falciparum* antigenicity among other factors led to emergence of drug-resistant strains of *P. falciparum* against virtually all commonly used antimalarial drugs [12-15]. The *in vivo* sensitivity assessment of individual drugs will amount to monothera-py, which is unrealistic due to ethical issues arising from current combination therapy guidelines. In addition, reduced *in vitro* sensitivity usually precede *in vivo* treatment failures and resistance; both complementing each other. *In vitro* assessment exposes malaria parasites directly to known antimalarial drug concentrations without confounding host factors; thus defining true resistance [11]. CQ resistance is associated with a specific point mutation at various codons in *P. falciparum* CQ resistance transporter (*Pfcrt*) [16,17] and modulates by mutations in the *P. falciparum* multidrug resistance locus 1 (*Pfmdr1*) [18]. The mutations at codons 76 (*Pfcrt* K76T) and 86 (*Pfmdr1* N86Y) are most important for *Pfcrt* and *Pfmdr1*, respectively [19,20]. Hence, genotyping of these genes is often used for resistance assessment.

In Nigeria, over-the-counter antimalarial drugs are readily available and are often used inappropriately [21,22] resulting in the exposure of malaria parasites to sub-lethal concentrations of drugs. In the present study, the *in vitro* sensitivity of AQ and CQ against *P. falciparum* clinical isolates from Northeast Nigeria was assessed.

## 2 Materials and methods

### 2.1 Study locations

*P. falciparum* Clinical Isolates (PfCIs) were obtained from two northeast towns, Azare and Maiduguri. Azare, Bauchi State (11^o^40’35” N and 10^o^11’41” E) [23], is on the Sudan Savannah belt of Northeast Nigeria with an estimated average annual rainfall of 768 mm and average annual low and high temperature of 18.8 and 34.1oC, respectively [24]. The last National Census put the Azare population at 110,452 [25]. Maiduguri (11^o^50’47” N and 13o9’36” E) [23], the capital of Borno State, is located in the Sahel Savannah of Northeast Nigeria with an estimated total annual rainfall of 613 mm. The average annual low and high temperatures are 18.2 and 33.3 ^o^C, respectively [26]. The Maiduguri population was 732,696 during the last National Census [25]. The two study areas are malaria-endemic, with year-round transmission with peak intensity during the rainy season (July – September).

### 2.2 Subject enrolment and collection of PfCIs

Subjects were enrolled at the Federal Medical Centre (FMC), Azare; University of Maiduguri Medical Centre (UMMC), Maiduguri and the University of Maiduguri Teaching Hospital (UMTH), Maiduguri. The ethical approval for this study was obtained from the Ethics Committee of FMC and UMTH. Initial malaria screening was done using microscopy and Giemsa-stained thick and thin blood smears prepared from finger-pricked blood samples [11]. Between September and November 2012, individuals that were confirmed as having acute uncomplicated *P. falciparum* malaria with percentage parasitaemia of 0.3 – 1.0 % were requested to participate. Following full informed consent, 100 μl of blood was aseptically collected into a sterile heparinized capillary tube and was quickly dispensed into a sterile 1.5 ml eppendorf tube containing 900 μl of incomplete RPMI 1640 media to give 1 ml of blood-medium-mixture (BMM). The BMM was kept in an ice pack and transported to the laboratory within 6 hrs after collection [27]. The *in vitro* assay was conducted in batches of 5 – 10 isolates; uniformity of the assays was maintained for all the batches.

All the patients were treated with either artemether-lumefantrine or artesunate-amodiaquine and followed up for 14 days using the standard *in vivo* protocol. Treatment outcomes have been reported elsewhere [10].

### 2.3 Preparation of drug pre-dosed microtitre plates

AQ hydrochloride, CQ phosphate and 96-well microtitre plates (Nunc plate®) were procured from Sigma-Aldrich Corporation**,** Bangalore, India; stock and working concentrations of the drugs were prepared in distilled water. The wells in rows B – H of each plate were pre-dosed with ascending concentrations of AQ (5, 10, 20, 40, 80, 160 and 320 nmol/l) and CQ (20, 40, 80, 160, 320, 640 and 1,280 nmol/l) in duplicates. The wells in row A served as control without drug. The drug pre-dosed plates were air-dried in laminar flow hood, wrapped with aluminium foil and stored at 4 °C until use. Pre-dosed plates were prepared weekly and were used within 2 weeks after preparation. CQ-sensitive 3D7 and CQ-resistant Dd2 *P. falciparum* clones were used as references to test each batch of plates [27,28].

### 2.4 *In vitro* drug sensitivity assay

The *in vitro* sensitivity of AQ and CQ against the 90 PfCIs was assessed by the *in vitro* micro-test (Mark III) method [27,29]. Briefly, 50 μl of the BMM was dispensed into each well of the drug pre-dosed plates and the culture plates were incubated at 37 ^°^C (± 0.5 °C) for 24 – 30 hrs in a candle jar creating an atmosphere of relatively high CO2 and low O2. The test was considered valid when at least 10% of the parasites (> 20 schizonts with at least 3 nuclei per 200 asexual parasites) attained schizont stage. Then, the supernatant from each well was gently removed and a thick smear prepared from the sediment on one slide for each column. The smears were air-dried, dehaemoglobin-ized and stained with 5% Giemsa stain for 15 min. Schi-zont maturation was estimated by counting the number of schizonts with at least 3 nuclei against 200 asexual ® parasites using X100 objective lens of an Olympusbinocular light microscope. The schizont maturation inhibition (SMI) was determined [27,29,30]. The experiment was repeated three times for each isolate and the mean values of valid results taken.

### 2.5 Data analysis

Data analyses were performed using SPSS version 15.0 and significance inferred at p < 0.05 [31]. The IC50 (drug concentration that inhibited schizont maturation in 50 % of the parasite population) of AQ and CQ for individual PfCIs was determined using HN-NonLin Version VI.1 software [32] and expressed as geometric mean, 95% confidence interval and range. The cut-off values for *in vitro* AQ and CQ resistance were put at 80 and 160 nmol/l [27]. Proportions were compared using Chi-square tests and two -sample t-tests were used for comparison of means.

## 3 Results

### 3.1 Characteristics of the enrolled subjects

A total of 187 subjects were screened during the study period. Ninety fulfilled the inclusion criteria, were enrolled, and *P. falciparum* isolates collected from them. The characteristics of the enrolled 90 subjects are presented in Table 1.

**Table 1.** Characteristics of the subjects who met the inclusion criteria.

Parameters	Azare	Maiduguri	Total
Number Screened	72	115	187
Number Enrolled	30	60	90
Mean age (yrs)	27.2 (± 10.0)^a^	22.5 (± 9.8)^a^	24.4 (± 10.0)^a^
Sex (male : female)	18:12	39:21	57:33
Mean haematocrit (%)	35.9 (± 9.1)^a^	34.6 (± 7.2)^a^	34.9 (± 8.6)^a^
GM parasite density (/μl)	5889 (5187-6631)^b^	4210 (3460-4910)^b^	4900 (4057-5720)^b^

GM: Geometric mean; a Standard deviation in parentheses; b 95% Confidence interval in parentheses

### 3.2 *In vitro* sensitivity assay success rate

Valid assay outcome was observed for 80 of the 90 PfCIs giving a success rate of 88.9 % (80/90) with Azare PfCIs having 93.3 % (28/30) as against 86.7 % (52/60) for Mai-duguri PfCIs (χ^2^ = 0.35; df = 1; p = 0.486). Subsequent analyses were based on the 80 PfCIs with valid results. Of the 10 PfCIs with invalid results, 3 had contamination while 7 recorded insufficient schizont growth (3.5 – 6.0 %) following 30 hrs of incubation.

### 3.3 *In vitro* AQ sensitivity against PfCIs

**Figure 1.**
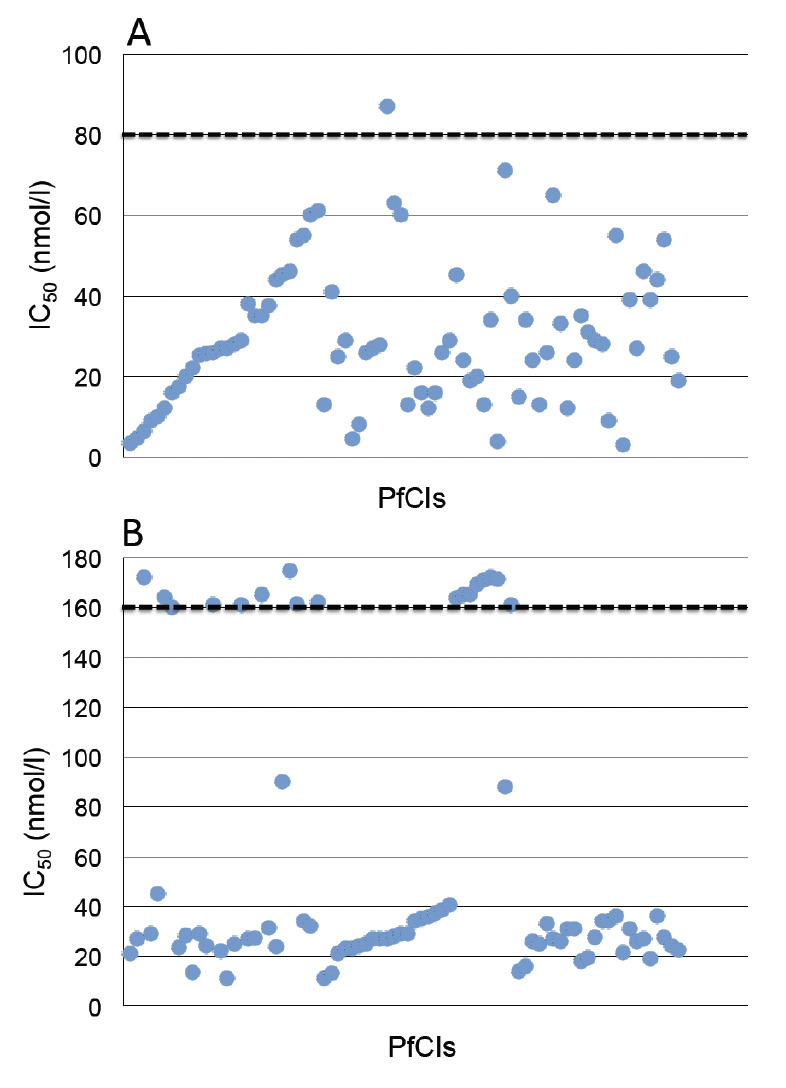
Scatter plot of amodiaquine IC50 (A) or chloroquine (B) for individual *P. falciparum* clinical isolates. The dotted lines represent the resistance cut-off lines.

The geometric mean (GM) IC50 of AQ was 24.2 nmol/l (95% CI, 10.5-49.6nmol/l) and was similar (p = 0.922) among the PfCIs from Azare and Maiduguri (Table 2). Of the 80 PfCIs, only one had AQ IC50 above 80 nmol/l (Figure 1A) giving *in vitro* sensitivity of 98.8% (79/80; Table 2). The only AQ-resistant isolate (IC50 = 87 nmol/l) was obtained in Maiduguri from a subject with 0.7 % para-sitaemia. In addition, 18 (22.5 %) isolates had an IC50 within the upper half (40 – 80 nmol/l) of *in vitro* sensitivity (Figure 1A).

**Table 2.** *In vitro* sensitivity of amodiaquine and chloroquine against *P. falciparum* clinical isolates (PfCIs).

Drug	Azare	Maiduguri	Total	p value
*Amodiaquine (AQ)*				
Number of PfCIs	28	52	80	-
GM (95% CI) [nmol/l]	23.9 (12.6-45.0)	23.5 (9.6-49.9)	24.2 (10.5-49.6)	0.922
Range IC50 (nmol/l)	3.5 – 61.0	3.0 – 87.0	3.0 – 87.0	-
Number of IC50 > 80 nmol/l	0	1	1	-
*In vitro* sensitivity (%)	28 (100.0)	51 (98.1)	79 (98.8)	0.497
*Chloroquine (CQ)*				
Number of PfCIs	28	52	80	-
GM (95% CI) [nmol/l]	48.2 (43.3-52.7)	35.4 (30.1-39.6	39.5 (34.5-49.6)	0.085
Range IC_50_ (nmol/l)	11.0 – 174.6	11.0 – 172.2	11.0 – 174.6	-
Number of IC_50_ > 160 nmol/l	9	8	17	-
*In vitro* sensitivity (%)	19 (67.9)	44 (84.6)	63 (78.8)	0.081

GM: Geometric mean; CI: Confidence Interval; 3D7 AQ = 16.6 nmol/l; CQ = 22.4nmol/ l; Dd2 AQ = 29.7nmol/l; CQ = 182.4nmol/l.

### 3.4 *In vitro* CQ sensitivity against PfCIs

CQ had GM IC50 of 39.5 nmol/l (95% CI, 34.5 – 49.6 nmol/l) which was similar among PfCIs isolated in Azare and Maiduguri (p = 0.085) as shown in Table 2. Seventeen (17) of the PfCIs had IC50 values above 160 nmol/l (Figure 1B) giving *in vitro* sensitivity of 78.8% (63/80). The sensitivity was similar among Azare (67.9 %; 19/28) and Maiduguri (84.6 %; 44/52) PfCIs (χ^2^ = 3.05; df = 1; p = 0.085; Table 2). The GM IC50 of the 17 CQ-resistant isolates was 165.8 nmol/l (95% CI, 163.4 – 168.3 nmol/l) with a mean percentage parasitaemia of 0.8 ± 0.3 %. Two additional isolates had an IC_50_ within the upper half of CQ *in vitro* sensitivity (Figure 1B).

## 4 Discussion

In the present study, the *in vitro* sensitivity of *P. falcipa-rum* clinical isolates to AQ and CQ was assessed in Northeast Nigeria. In this arid part of Nigeria information about the *in vitro* sensitivity of *P. falciparum* is scanty. Efforts to find results from similar studies, since the 2004 change of malaria treatment policy from CQ to ACTs, were not successful.

The *in vitro* assay success rate of 88.9% recorded in this study is significantly higher than 58.1% (36/62) and 64.5% (40/62) recorded in Southwest Nigeria [33]. However, it is comparable to 69.4% reported in Bangui, Central African Republic [34]. Several factors have been identified that affect the success rate of *in vitro* tests. These may include the level of parasitaemia, contamination, handling, storage and transportation of samples and the presence of drug residues in the blood [29]. Careful selection of subjects, with optimum parasitaemia and who had not taken antimalarials prior to enrolment, might have contributed to the high level of success in the present study.

Monodesethylamodiaquine is the principal metabolite of AQ in humans. However, *in vitro* assessments are often conducted with either the parent AQ [33,35] or the metabolite [36]. In our study, the parent AQ was used. AQ demonstrated adequate *in vitro* sensitivity against PfCIs from Northeast Nigeria, which emphasises the continuing relevance of the drug for the treatment of malaria. There is no previous *in vitro* data from the region; hence, this serves as baseline data for future reference. The sensitivity (98.8 %) recorded in this study is similar to 98.6 % reported in Northcentral Nigeria [37] but in sharp contrast to 61.0 % reported in Southwest Nigeria [33]. This contrast could be partly attributed to the fact that the Southwest isolates were collected during high CQ pressure and cross-resistance has been reported between AQ and CQ [38].

Owing to widespread resistance CQ has been replaced with ACTs in Nigeria for over a decade [9]. However, its re-introduction into the mainstay of malaria treatment and prevention has been predicted [39,40]. In the present study, CQ demonstrated higher *in vitro* sensitivity in Northeast Nigeria when compared to Southwest Nigeria [33,41]. Genotyping of biomarker (*Pfcrt*) of CQ resistance of the isolates would have provided additional explanation to support our findings. Interestingly, the present findings corroborate an earlier report of low prevalence of biomarkers of CQ resistance in the region [42]. The possibility of an indication that CQ sensitivity may be gradually returning to Northeast Nigeria following reduced drug pressure could be considered, however, the lack of similar studies in the same area would not permit this. Return of CQ sensitivity is attributed to re-expansion of a heterogenous population of susceptible parasites that persisted during the period when CQ was used [43].

## 5 Conclusions

The present study provides *in vitro* epidemiological data on sensitivity of PfCIs to AQ and CQ in Northeast Nigeria. The data showed that the isolates remain sensitive to AQ. The study also recorded a relatively high sensitivity of the isolates to CQ compared to previous studies in other parts of the country, perhaps, a prospect of return of CQ sensitivity following a period of official suspension of its use for malaria treatment.
